# Comparison of Cardiac Auscultation Features on Four Different Simulation Mannequins Performed by Pediatric Residents

**DOI:** 10.7759/cureus.45127

**Published:** 2023-09-12

**Authors:** Saud A Bahaidarah, Abdulaziz M Boker

**Affiliations:** 1 Pediatrics, Faculty of Medicine, King Abdulaziz University, Jeddah, SAU; 2 Clinical Skills and Simulation Centre, Faculty of Medicine, King Abdulaziz University, Jeddah, SAU

**Keywords:** auscultation accuracy, high fidelity mannequin, cardiac mannequin, cardiac auscultation, auscultation skill

## Abstract

Introduction: Cardiac murmurs are a common problem in pediatric clinical practice. Studies demonstrated low accuracy in detecting and diagnosing various cardiac murmurs at all levels of medical training. So, supplementary training methods started to evolve, including simulation for auscultation skills training. Over the years, mannequins have evolved with different types of technology. Therefore, we decided to compare cardiac auscultation accuracy among high-fidelity mannequins as the primary objective and compare the performance of various postgraduate-level residents as a secondary objective.

Method: Pediatric residents at King Abdulaziz University Hospital were given a lecture on the basics of cardiac auscultation and then requested to auscultate four mannequins, namely SimJumior® (Laerdal Medical, Stavanger, Norway), SimBaby™ (Laerdal Medical), Pediatric HAL® (Gaumard Scientific, Miami, FL, USA), and Cardiac Patient Simulator K-Plus (Kyoto Kagaku Co. Ltd., Kyoto, Japan). The accuracies of murmur type, diagnosis, and auscultation time were compared.

Results: A total of 56 pediatric residents were enrolled. Median murmur accuracy ranged from 50% to 53% (p-value 0.79), and median diagnosis accuracy ranged from 33% to 36% (p-value 0.77), with a nonsignificant difference between mannequins. Comparing resident levels in all mannequins, median murmur accuracy ranged from 49% to 56% (p-value 0.70), and median diagnosis accuracy ranged from 29% to 41% (p-value 0.09). While the median average auscultation time was between 41 and 50 seconds (p-value 0.34).

Conclusion: Auscultation skills can be taught through simulation on any mannequin used in this comparison, not necessarily the cardiac one. For better accuracy, future comparisons might include more advanced cardiac mannequins based on cardiac auscultation expertise (i.e., consultant level). The introduction of an auscultation program from the undergraduate level throughout the training process and monitoring of these skills are mandated.

## Introduction

Cardiac murmurs are seen in about 50% of children and are reportedly innocent murmurs [1]. Additionally, auscultatory skills should decrease the number of unnecessary referrals to cardiologists while, at the same time, not missing a life-threatening condition in congenital heart disease [2]. Cardiac auscultation is considered one of the basic skills that undergraduate medical students need to master during their medical school training. After graduation from medical school in an office-based practice, evaluation of physician competency showed suboptimal accuracy in diagnosing heart murmurs [3]. Therefore, studies tried to evaluate different categories of physicians and found the accuracy of cardiac auscultation below expectations [2,4-7]. This is due to multiple factors that might include busy clinics and a high volume of patients [8], especially with the availability of more sophisticated cardiac investigation that is easier to order but expensive, rather than spending more time in detailed appropriate auscultation, keeping in mind the unavailability of typical patients and respect for patients as factors affecting this skill's training [9]. Due to such shortcomings, supplementary methods of training evolved that included a self-directed cardiac auscultation program [5], the studying of and unlimited review of recorded heart sounds [10], and audio lectures demonstrating normal and pathological murmurs in addition to self-study via MPEG audio layer 3 (MP3) players [11].

All of these techniques showed an increase in the accuracy level of detecting heart murmurs. However, there are still difficulties in accurately detecting heart sounds in a patient, which most of the time depended on a student's performance on an audio or multimedia test [12], so simulation started to play an important role. Simulators have many advantages, including avoiding risks to patients and learners, practicing skills repeatedly, enhancing the transfer from the classroom to real situations, and standardizing learner assessment [13]. Collaboration is significantly better in a simulation environment compared to a traditional learning environment in baccalaureate pre-licensure nursing [14]. Also, adding clinically appropriate scenarios increases participants’ confidence [15]. Using simulation in training gives the learner the opportunity for assessment and ongoing feedback [16]. Auscultation accuracy is higher in students trained in cardiac patient simulators compared to real patient training with a more accurate examination of actual patients [17].

The Harvey mannequin (Laerdal Medical, Stavanger, Norway) is a full-sized computer-based mannequin that simulates 27 cardiac conditions and was first demonstrated in 1968. It was tested up until 1987 and introduced into five medical schools, training 208 medical students who performed better than their peers and trained on real patients with better confidence and ability to interpret findings at the bedside [18]. Another example is the Ventriloscope simulator (Lecat’s Ventriloscope® LLC, Tallmadge, Ohio, USA) that integrates pre-recorded sounds with a simulated patient to improve objective structured clinical examination (OSCE) [9]. An additional example of a cardiac auscultation simulator is the one developed by Takashina et al. in 1990, a human chest-size mannequin with prerecorded heart sounds and murmurs that could be heard in the corresponding auscultatory areas [19]. In high- and low-fidelity simulations (fidelity is the extent of the appearance and behavior of the simulator or simulation to match the simulated system), low fidelity is comparable or even superior to high fidelity regarding auscultation skills, possibly due to external distractors with high-fidelity mannequins [20]. So, with the presence of variable mannequins in the market that are used primarily for teaching cardiac examination and high-fidelity mannequins used for other simulation purposes, including cardiac murmurs, we decided to compare the accuracy of cardiac auscultation between these mannequins as a primary objective while also evaluating various residents' levels of auscultation accuracy as a secondary objective.

## Materials and methods

This is a cross-sectional intervention study conducted at the Clinical Skills and Simulation Center (CSSC) at King Abdulaziz University, Jeddah, Saudi Arabia. Various levels of pediatric residents in the Saudi Board of Pediatrics under the Saudi Commission for Health Specialties (SCFH) training at the King Abdulaziz University Hospital (KAUH) were enrolled. After receiving approval from the Unit of Bioethical Research committee at KAUH (approval no. 411-20), residents attended a lecture that included the basics of cardiovascular auscultation, an explanation of various types of murmurs, and how to differentiate between them via a video demonstration of auscultation areas followed by an audio demonstration of heart sounds and multiple types of heart murmurs. Voluntary attendance and participation were considered consent.

The available pediatric mannequins in CSSC from different vendors were used, namely SimJumior® (Laerdal Medical, Stavanger, Norway), SimBaby™ (Laerdal Medical), Pediatric HAL® (Gaumard Scientific, Miami, FL, USA), and Cardiac Patient Simulator K-Plus (Kyoto Kagaku Co. Ltd., Kyoto, Japan). These mannequins are used for the purpose of cardiopulmonary resuscitation simulation training, except K-Plus, which is a cardiac patient simulator. All of these mannequins contain various normal and abnormal cardiac sounds for auscultation. All the monitors connected to these mannequins were removed to avoid any distractions.

The following types of murmurs are included in this study: normal heart sounds (no murmur), ventricular septal defect, pulmonary stenosis, aortic stenosis, aortic regurgitation, and patent ductus arteriosus. These are common congenital heart diseases and are frequently used in the literature, in addition to being available on the mannequins at the CSSC. Each candidate documented on a form the type of murmur, diagnosis, and auscultation time, which is the time needed for auscultation, starting from the moment of putting the stethoscope on the mannequin to auscultate and ending by removing it. The candidate calculated the auscultation time using the stopwatch features on their mobiles. Two pediatric cardiology consultants examined all the mannequins for the type of murmurs. Their findings were compared with the built-in labeling on these mannequins, and they formulated a diagnosis list for each, which was considered while entering the collected data. We did a pilot study with four pediatric specialists and four house officers (the last year of medical school); they were asked to auscultate each mannequin and fill out the data collection sheet. From their results, organization time and flow were obtained. All the residents received the exact instructions, which included: (1) a scenario of an asymptomatic patient who came to the clinic for cardiac evaluation; and (2) no second-time auscultation after moving to the next murmur.

Each candidate had rotated on all mannequins. The K-Plus and SimBaby mannequins have five murmurs to auscultate, while the SimJunior and Pediatric HAL mannequins have four. So, eventually, each resident auscultated and recorded 18 murmurs. During the auscultation, all monitors and speakers were removed. Other sounds produced by the mannequin, such as respiratory sounds, were suppressed.

Statistical analysis

Data were analyzed using SPSS Statistics version 26 (IBM Corp., Armonk, NY, USA). Categorical variables were expressed as numbers, while numeric variables were presented in median and range (minimum to maximum). Comparisons between groups were tested using the nonparametric Kruskal-Wallis test (multiple comparisons were not performed because the overall test did not show significant differences across samples). Statistical significance was considered if p <0.05. Accuracy was calculated for the type of murmur and diagnosis based on the residents' answers as a percentage of correct answers to the total auscultation number and categorized per their postgraduate level for each murmur and mannequin. The averages of murmur accuracy, diagnosis accuracy, and auscultation time were calculated for each group of residents on each of the mannequins. Groups were compared by calculating the median and range (minimum and maximum) of each group's accuracy for the type of murmur and correct diagnosis for each mannequin. The average type of murmur and diagnosis accuracy of the whole group (residents) were calculated for each mannequin, and by using the median, these mannequins were compared. The median was chosen for comparison due to non-normally distributed variants and non-equal small numbers in each group. Auscultation time was recorded in seconds and averaged per candidate per mannequin. Then time was averaged per group for all mannequins, and groups were compared using the median and range (maximum and minimum) of auscultation time.

## Results

Demographics

A total of 56 pediatric residents from all levels of training, 11 from postgraduate year 4th level (PGY4), 13 from PGY3, 17 from PGY2, and 15 from PGY1, performed auscultation, with females representing 66% (37 candidates) and males at 34% (19 candidates).

Mannequin comparison

Initially, the calculation of accuracies for murmur and diagnosis, besides averages of auscultation time for each resident's level for each mannequin, was done (Table 1). Murmur median accuracy for all the residents per mannequin ranged from 50% to 53% with a statistically nonsignificant difference (p-value 0.79), while median diagnosis accuracy was lower, ranging between 33% and 36% with a statistically nonsignificant difference (p-value 0.77) between the mannequins (Table 2).

**Table 1:** Averages of murmur accuracy, diagnosis accuracy, and auscultation time among residents of different postgraduate levels on different mannequins PGY: Postgraduate year ^*^Murmur accuracy: Median (minimum-maximum) and is the percentage of the correct answers ^**^Diagnosis accuracy: Median (minimum-maximum) and is the percentage of the correct answers ^+^Average time median (minimum-maximum) and is in seconds ^$^p-value < 0.05 is considered significant

Accuracy on mannequins	PGY4 (n=11)	PGY3 (n=13)	PGY2 (n=17)	PGY1 (n=15)	p-alue^$^
K-PLUS					
Murmur accuracy (%)^*^	60 (20-100)	40 (20-100)	40 (20-100)	60 (0-100)	0.307
Diagnosis accuracy (%)^**^	40 (20-80)	40 (20-60)	20 (0-80)	40 (0-80)	0.103
Average time, seconds^+^	44 (18-81)	54 (19-90)	42 (11-98)	56 (29-125	0.726
SimBaby					
Murmur accuracy (%)	40 (20-80)	40 (20-80)	40 (20-80)	60 (0-100)	0.841
Diagnosis accuracy (%)	40 (20-60)	40 (20-80)	20 (0-80)	20 (0-60)	0.154
Average time, seconds	33 (12-66)	60 (30-83)	60 (8-103)	41 (20-100)	0.160
SimJunior					
Murmur accuracy (%)	75 (0-100)	50 (25-75)	75 (0-100)	50 (0-75)	0.135
Diagnosis accuracy (%)	50 (0-75)	25 (0-50)	25 (0-100)	25 (0-100)	0.345
Average time, seconds	40 (23-86)	49 (19-112)	52 (12-79)	45 (35-85)	0.250
Pediatric HAL					
Murmur accuracy (%)	50 (0-100)	50 (25-100)	50 (25-100)	50 (0-100)	0.626
Diagnosis accuracy (%)	50 (0-75)	50 (25-100)	50 (25-100)	25 (0-100)	0.280
Average time, seconds	32 (19-53)	40 (19-98)	42 (10-72)	49 (21-95)	0.115

**Table 2:** Total averages of murmur accuracy and diagnosis accuracy in different mannequins

Accuracy	K-Plus	SimBaby	SimJunior	Pediatric HAL	p-value
Murmur accuracy					
Median (minimum-maximum)	53 (44-59)	50.5 (46-53)	50 (38-59)	52 (50-59)	0.790
Diagnosis accuracy					
Median (minimum-maximum)	34.5 (30-45)	36 (27-43)	33.5 (23-39)	35.8 (34-47)	0.777

Resident-level comparison

Comparing the residents' postgraduate levels in all mannequins (Table 3), median murmur accuracy ranged from 49% to 56% (p-value 0.70), and median diagnosis accuracy ranged from 29% to 41% (p-value 0.09). The median average auscultation time was between 41 and 50 seconds (p-value 0.34), with PGY2 being the shortest and PGY1 being the longest.

**Table 3:** Total averages of murmur accuracy, diagnosis accuracy, and auscultation time as per postgraduate levels PGY: Postgraduate year ^*^Murmur accuracy: Median (minimum-maximum) and is the percentage of the correct answers ^**^Diagnosis accuracy: Median (minimum-maximum) and is the percentage of the correct answers ^+^Average time: median (minimum-maximum) and is in seconds ^$^p-value < 0.05 is considered significant.

Accuracy	PGY4 (n=11)	PGY3 (n=13)	PGY2 (n=17)	PGY1 (n=15)	p-value^$^
Murmur accuracy (%)^*^	56 (39-72)	49 (39-72)	52 (22-77)	49 (21-64)	0.708
Diagnosis accuracy (%)^**^	41 (25-56)	37 (27-56)	32 (10-61)	29 (15-54)	0.090
Average time, seconds^+^	41 (27-66)	50 (31-80)	47 (14-80)	50 (30-82)	0.337

## Discussion

Since 50% of children report innocent murmurs [1], and congenital heart disease incidence is near 1% of live births, the importance of cardiac auscultation competency is emphasized during pediatrician training. One of the most common referrals to pediatric cardiology clinics is an evaluation for a murmur [5]. Studies done to evaluate various levels of physicians and specialties showed low auscultation accuracy [2-7]. So, studies started to compare different training or teaching methods of auscultation. Mahnke et al. showed improvement in auscultation skills by using a self-directed auscultation program in relation to an outpatient rotation in a cardiology clinic [5]. Also, searching for structured auscultation training, as in a 1993 United States survey, showed no structured teaching of cardiac auscultation in three-fourths of internal medicine programs and two-thirds of cardiology fellowships [21]. In the United Kingdom medical schools, heart sound simulators are used as an introduction to heart murmurs rather than a repetitive tool to reach a competent level, and at the same time, the effectiveness of such a method of teaching in the clinical examination was not measured [22]. Our group showed low accuracy in detecting the correct type of murmur (around 50%), which means there is a high chance of missing an important murmur with a significant clinical impact. At the same time, they showed a lower diagnosis accuracy (around 34%). Both low accuracies have been established by Gaskin et al. (via simulators); they demonstrated low accuracy in auscultation in pediatric and internal medicine residents to be 36% to 46% [2]. This is also comparable to Haney et al. (auscultation of real patients), who determined auscultation accuracy of 20% to 54% among internal medicine and family medicine residents [3], which is considered slightly higher than Mangione et al. (determined via pre-recorded heart sounds), whose group accuracy was not more than 19% in the auscultation of 12 cardiac events [21]. So in the last decades, several tools have emerged to facilitate the learning of cardiac auscultations, including auscultation training programs in undergraduate or postgraduate training that include electronic stethoscopes, audiovisual instruments, and multimedia [9] to improve auscultation accuracy.

Guarda et al. showed that after training on the Student Auscultation Mannequin (Cardionics Inc., Webster, TX, USA), the rate of correct auscultation improved from 33% and 40% to 78% in internal medicine residents [4]. This improvement in low accuracy was also seen by Perlini et al.; students and residents who had 11% and 16% accuracy, respectively, had an improvement up to 72% and 76%, respectively, after 10 hours of simulation-based practice [9]. Other observations indicated that there was no difference in the level of accuracy between the two levels of medical students and residents [9]. This is seen in our group; despite the different levels of PGY with various exposure to cases and cardiology rotation, there is no significant difference between the junior level (PGY1, PGY2) or senior level (PGY3, PGY4) of training regarding the accuracy of murmur type and diagnosis. For auscultation time, PGY1 showed the longest time in the group while PGY4 was the shortest, but statistically, this variation is nonsignificant. Vukanovic-Criley et al. showed that there is no improvement in cardiac examination skills from the medical student level to the residency level [23], which might necessitate adding an auscultation curriculum to the residency training program or even from the undergraduate level with correlation to real patient auscultation [9,17,24].

Simulation is an educational modality that can be used for auscultation as a technique that allows interactive and immersive activity by recreating all or part of a clinical experience without exposing patients to any risks [13]. The advantages of using mannequin-based computer simulators include determining the learning needs, applying the experience or training in a controlled environment with unlimited repetition of a procedure till mastery is achieved, reinforcement of standard guidelines, evaluation, and feedback with no issue of patient safety or confidentiality, and helping when there is an ethical concern about using a technique on real patients [16,22,25]. The concept of a part-task trainer as a clinical simulator started to evolve with the Harvey cardiology simulator [13], which was first demonstrated in 1968 and is capable of demonstrating various cardiac examinations with a broad spectrum of cardiac diseases [18]. The other cardiac mannequins started to develop similarly, such as the one designed by Takashina et al. in 1990 [19] and the Ventriloscope simulator [9]. Other mannequins were developed for medical training in the early 1960s by Resusci®-Anne (Laerdal Medical) that came ahead of computer-controlled mannequins [18]. Then, the term fidelity as a common industry term started to evolve, describing the degree of realism and technical complexity of models [25], reaching high-fidelity mannequins with physiologic and non-physiological programming [26]. These might have heart sounds that are used during medical training. So, we used the available mannequins in CSC at KAUH.

Pediatric HAL® S3005 is a wireless and tetherless five-year-old patient simulator that trains teamwork and patient care skills through hands-on exercise from nursing to emergency care [27]. SimBaby™ is a tetherless simulator designed to help healthcare providers effectively recognize and respond to critically ill pediatric patients. It represents a nine-month-old pediatric patient and provides a highly realistic mannequin that meets specific learning objectives focusing on initial assessment and treatment, while SimJunior® represents a six-year-old boy that simulates a wide range of conditions, from a healthy, talking child to an unresponsive, critical patient with no vital signs [28]. These three mannequins, besides all the other features, contain normal and abnormal heart sounds, unlike Cardiac Patient Simulator K-Plus, which is meant to teach cardiac examination with auscultations of various heart sounds and another component of cardiac examination [29], which has the highest median accuracy in the type of murmur detected by our group. The accuracy of detecting the diagnosis was highest in SimBaby™. In the end, all these mannequins showed nonsignificant differences regarding the accuracy of murmur auscultation and diagnosis, which means teaching auscultation on each is the same as there is no difference in the type of murmur or arriving at the diagnosis. All the attached monitors, speakers, and respiratory sounds were removed or suppressed during the auscultation to prevent distraction [20]. The candidates were instructed that once done with a murmur auscultation, they could not go back to re-auscultate. However, in the usual daily practice, re-auscultation occurs for data or patient chart completion. However, we prohibited it for standardization between all candidates, keeping respect for time and organization in mind. 

The small number of residents in the groups is considered one of the limitations of this comparison, along with the lack of a control group to compare the mannequins with the effect of pre-auscultation teaching sessions. Due to the limited number of heart murmurs on the mannequins, we could not compare non-pathological to pathological murmurs, and not all types of diagnoses existed in all mannequins; for that reason, the accuracies were compared as all murmurs in the same mannequin instead of each type of murmur in each one. Despite the accuracy of auscultation being comparable to what has been published before at the postgraduate level, a higher level of auscultation expertise at the pediatric or adult cardiology consultant level might show the differences between these mannequins.

## Conclusions

Teaching auscultation skills through simulation can be performed on any mannequin used in this study, not necessarily the cardiac mannequin. Any mannequin with heart sound features can be used, which enables maximum utilization of the simulation mannequins with limited resources to have a dedicated cardiac simulator. The low accuracy of heart murmur auscultation and consequent diagnosis at various levels of pediatric residency training enlightened the need for an auscultation program from the undergraduate level and the monitoring of these skills until the advanced level of residency training. Future research comparing more advanced cardiac system examinations apart from auscultation skill and high-fidelity mannequins by cardiac auscultation experts (i.e., cardiology consultant level) might give better accuracy and comparison results.
